# Commitment & cooperation in social dilemmas with diverse individual preferences: An agent-based modeling approach

**DOI:** 10.1371/journal.pone.0327131

**Published:** 2025-07-11

**Authors:** Zheng Jiang, Luzhan Yuan, Wei Wang, Gaowei Zhang, Yi Wang

**Affiliations:** 1 School of Artificial Intelligence, Beijing University of Posts and Telecommunications, Beijing, China; 2 School of Computer Science, Beijing University of Posts and Telecommunications, Beijing, China; Sadat Academy for Management Sciences, EGYPT

## Abstract

Social dilemmas often arise when the need for cooperation conflicts with individuals’ incentives to act in self-interest, potentially undermining collective well-being. Prior literature shows that some mechanisms, e.g., commitment, could give rise to cooperation. However, participants’ diverse propensities to cooperate may limit such mechanisms’ effectiveness. Thus, we bring individual differences in their propensities to cooperate into the reasoning of long-term social dynamics of cooperation through an agent-based modeling (ABM) approach. Our results suggest that commitment may still guarantee cooperation when individuals have different propensities to cooperate but have weaker effects, and the setups of commitment are also important. Our study highlights the importance of integrating individual preferences in analyzing collective dynamics of a population consisting of individuals of heterogeneous characteristics, thus offering implications to facilitate cooperation in real-world online social production. The model and its implementation also form the foundation for supporting decision-makers in forming decisions of facilitating cooperation via commitment mechanisms.

## Introduction

Interpersonal cooperation is a fundamental component of many modern social and economic activities. However, cooperation neither emerges from nothing nor maintains and diffuses automatically [1–3]. Literature has shown that being cooperative is not an arbitrary personal choice but could be deliberated strategic behaviors in social cooperation [4]. When individual members decide to maximize their own short-term benefits independently, *social dilemma*, in which socially optimal could never be achieved, can happen [5]. Nobel Laureate Elinor Ostrom pointed out, in the collective actions driving social production, if many choose to be uncooperative, cooperators’ risk would significantly increase and become more doubtful of continuing to be cooperative [6], eventually resulting in a *tragedy of commons* [7] where no one cooperates.

Fortunately, people have found various mechanisms, such as incentives, social norms, etc., to resolve social dilemmas and develop, maintain, and enforce cooperation [8, 9]. Commitment is one such mechanism. Literature in multiple disciplines has confirmed commitment’s positive effects on solving social dilemmas and guaranteeing cooperation from both theoretical and empirical perspectives [10–13]. However, most extant literature on the interrelationship between commitment and cooperation explicitly or implicitly assumes a homogeneous population, neglecting individual preferences. I.e., they assume all members have the same propensity to be cooperative in interpersonal interactions. In fact, such an assumption may not always hold. Individual differences in their willingness to cooperate are consistently reported to have significant impacts on their cooperative behaviors [14, 15]. Besides, modern work organizations are increasingly globally distributed, their different backgrounds and the lack of collocated context could lead to much more diverse individual preferences in cooperative behaviors [16, 17], amplifying such differences’ impacts in cooperation with the presence of the commitment mechanism. Moreover, such personal-level differences also have complex and profound effects on the outcomes of cooperation in addressing social dilemmas. As Maxwell & Oliver pointed out in their influential book [18], even a small proportion of people with heterogeneous characteristics in a group, e.g., highly-motivated, could lead to outcomes that would be impossible to obtain with groups with strictly homogeneous characteristics.

Therefore, there is an imperative to conduct a thorough theoretical exploration of how diverse individual preferences influence the effects of commitment on solving social dilemmas and improving cooperation. We thus have the first research questions.

$RQ_{1}$: *Does diverse individual preferences influence the development of cooperation with the commitment mechanism?*

If the diverse individual preferences’ effects could be confirmed, we would like to examine two deeper characteristics of such effects. First, since prior literature [11] shows that the effects of commitment are largely determined by its setups, we would like to examine how these setups work when introducing individual preferences in cooperation; thus, we have the second research question:

$RQ_{2}$: *How do different setups of the commitment influence the cooperation of people of diverse individual preferences?*

Moreover, the development of cooperation with our model is a dynamic process, i.e., the effects of commitment mechanism may take some time to become stable. Reaching stability quickly would save individuals’ much effort in trial and error, and help establish behavioral conventions [19]. Therefore, we shall also pay attention to the process itself. Thus, our last research question is as follows.

$RQ_{3}$: *What are the impacts of individuals’ diverse preferences on the dynamic process of cooperation development, particularly in terms of efficiency and long-term stability?*

This article reports our efforts in answering the above research questions through the agent-based modeling (ABM) technique [20, 21] (Research method: Agent-based modeling (ABM)). Leveraging game theory, we built a model to simulate how individuals (*agents*) with different preferences in cooperation make strategic decisions in interacting with other members in a fixed population, with *commitment* as a type of strategies in dyadic interactions yet publicly-visible for all members (The ABM model). We then allowed the agents to interact with each other in a discrete event simulation setting and experimented with a wide range of model parameters, which enabled us to explore the long-term dynamics of cooperation(Simulation experiment design). Our experiments have yielded rich results and findings when considering individual preferences (Results & findings). We further discussed our work’s theoretical and practical implications, as well as its potential in supporting decision-making of social collaboration (Discussion).

Our work makes several contributions to the literature. First, our modeling and experiment characterized the dynamic interrelations of cooperation with individual preferences being explicitly considered, which extends the existing theoretical knowledge. Second, the results and findings have immediate implications for informing people committed to addressing social dilemmas, particularly decision-makers, in designing and implementing commitment mechanisms by calling for their intention to individual differences in the propensity to cooperate. Third, the agent-based model we developed features the innovative integration of behavioral theories, analytical models, and dynamic processes to model people’s complex decision-making and behaviors. Its highly extensible design and implementation make it could serve as the foundation for reasoning various social dilemmas in social interactions, thereby supporting key decision-making in multiple scenarios.

## Background & related work

### Social dilemmas with individual preferences

Social dilemmas have been extensively studied as a framework for understanding the tension between individual self-interest and collective well-being. Classical models, such as the Prisoner’s Dilemma and Public Goods Game, provide fundamental insights into cooperation dynamics, focusing on how individuals balance incentives to cooperate or defect. Previous studies have extensively explored cooperation in homogeneous populations, where all agents share similar preferences and behavioral tendencies [22, 23]. These studies demonstrate that repeated interactions and mechanisms like reciprocity can stabilize cooperation under certain conditions. However, more recent research has shifted focus to heterogeneous populations, where individuals differ in their preferences, strategies, and incentives, leading to more complex dynamics [24, 25]. Variations in player preferences, such as differences in reward matrices, interaction flow, and opponent composition, have been shown to significantly influence cooperation outcomes in the Prisoner’s Dilemma [26]. Research has also demonstrated that heterogeneity in individual payoffs can affect cooperation in evolutionary games, revealing that diverse preferences can stabilize cooperative behavior under specific conditions [27]. Inequality in endowments and productivities has been found to impact group coordination in threshold public goods games, where mismatched contributions hinder cooperation, while aligned heterogeneity fosters it [28].

Such diversity has been shown to significantly impact the dynamics of cooperation, especially in simpler models without additional mechanisms like commitment. However, despite the progress in modeling heterogeneity in simpler settings, the interplay between individual diversity and external mechanisms, such as commitment, remains underexplored. Our study builds on these foundations by integrating heterogeneous preferences into a model with commitment mechanisms, providing new insights into how diverse individual characteristics influence the stability of cooperation in social dilemmas.

### Commitment & cooperation

Commitment is a frequently used mechanism for promoting and maintaining cooperation. Its effectiveness has been confirmed by both theoretical and empirical literature from multiple domains [10–13]. Compared with other mechanisms, it has multiple advantages, particularly in contexts where transparency is high. First, one key benefit is its relatively low implementation and enforcement cost [29]. For instance, a commitment mechanism can be initiated through a simple declaration of intent within a transparent system. Its enforcement is inherently ensured by the visibility of actions, as the community collectively monitors and evaluates individual contributions. Second, it could be conveniently combined with other mechanisms. Multiple types of punishment could be a part of it since enforcing commitment often directly incurs some punishment [30]. Third, commitment mechanisms are often analytically tractable so that we can evaluate them in an analytical framework before their implementations, avoiding unfavorable consequences in their actual launching.

However, researchers seldom put individual preferences in cooperation into consideration when investigating the interrelationship between commitment and cooperation. According to Weber *et al*.’s appropriateness framework, decision makers’ individual differences influence how they interpret the situation and how perceptions of the situation impact their choices of behavioral decisions [31, 32]. For instance, some participants may always cooperate no matter what cost they would pay for it because being cooperative has some important personal value to them [33]. Others may always defect even when there are potential penalties as the consequences of their unfavorable behaviors [34]. Without considering individual preferences, it would seriously threaten the validity of the theoretical insight of related research work and the real-world applicability. For example, Han *et al*.’s theories [11, 30] often have difficulties explaining uncooperative behaviors’ persistence. The real-world effectiveness of the corresponding mechanisms could also be questionable. Our study is designed to fill such a research gap exactly.

## Research method: Agent-based modeling (ABM)

We choose agent-based modeling as the main research method to explore the role of individual preferences in the dynamics of cooperation, with the presence of commitment. ABM is a well-established yet evolving approach to modeling complex systems consisting of interacting, autonomous agents representing humans and nonhuman subjects [35]. In ABM, agents behave according to predefined rules in interactions with other agents, which may in return change their behaviors. By modeling a diverse set of agents, we could observe individual agents’ behavioral adaptations as well as the dynamics of the system as a whole. ABM thus enables us to analyze patterns, structures, and dynamics that were not explicitly programmed into the models but arise through the agent interactions [36].

Moreover, ABM also forms the foundation for developing sophisticated decision support systems [37]. By modeling and simulating actors’ characteristics and behaviors in complex social systems, ABM could inform decision-makers future dynamics, and let them experiment with different interventions and settings in a simulated environment, to identify optimal decisions. For example, Sueyoshi & Tadiparthi designed an ABM-based software to explore new trading strategies in a competitive electricity trading environment [38]; Chesney *et al*. applied ABM in creating a shadow account to help external stakeholders evaluate corporate disclosures and corporate action [39].

In our study, by learning and adapting their behaviors, agents may choose to be cooperative and uncooperative, jointly determining the overall cooperation dynamics in the fixed population. First, the dynamics of cooperation are generated bottom-up, realizing the leap from micro personal motives and actions to macro system behaviors [40]. Second, individual preferences and commitment drive individual behaviors together, implying the model needs to combine multiple theories to be a valid representation of reality. Finally, the system-level attributes and dynamics cannot be intuitively predicted based on rules for individual actions because the multiple forces affecting behaviors may work in various directions. Obviously, it is appropriate for ABM to be the main research strategy of our study.

The agent-based model described in this article simulates the behaviors of members of diverse preferences on cooperation in a fixed population during their interactions with other members. Doing so enabled us to understand the long-term dynamics of cooperation from both individual and population levels and how individual preferences and commitment shape cooperation in the population. We are going to introduce its details in this section.

## The ABM model

### Theoretical foundations

Properly specifying agents’ decision-making and behaviors is critical for an ABM model. Their decision-making and behaviors shall be grounded by theoretical knowledge and/or empirical observations.

#### A game extending social dilemma with commitment.

To incorporate commitment into social dilemmas, we extended the classic framework and adopted the model proposed by Han [30] to examine cooperation dynamics within such scenarios. Rather than applying the model to a specific real-world context, this study aims to explore the theoretical foundations of the model and provide valuable insights into its broader implications. Since proposing commitment is an action, it could be viewed as a part of strategies. A commitment, once made, may not always be fulfilled later. Thus, we need two extra strategies. One is for making a commitment and fulfilling it, and another is for making but not fulfilling. In addition, some members may wait for other parties’ commitment as the prerequisite of their own cooperative behaviors; we need another extra strategy. Therefore, there are potentially five strategies. That is similar to what Han [30] proposed. We reuse and slightly revise their game structure as follows:

Compared with the classic prisoner’s dilemma, the extended game (Table 1) incorporates five strategies: the classic strategies *C* (cooperate) and *D* (defect), along with three additional strategies, *COM_C*, *COM_D*, and *COM_O*, which introduce commitment mechanisms. **Parameter Definitions**

- **T**: The payoff a player receives for defecting against a cooperator. In a typical Prisoner’s Dilemma, *T* is the highest individual payoff (i.e., *T* > *R*).
- **R**: The payoff both players receive if they mutually cooperate.
- **P**: The payoff both players receive if they both defect.
- **S**: The payoff to a cooperator when the opponent defects. Typically, S is the lowest payoff in the classic Prisoner’s Dilemma ordering (*T* > *R* > *P* > *S*).
- ***c***: A cost paid by an agent who establishes a commitment.
- ***w***: An additional term that modifies payoffs when one side promises cooperation but defects (i.e., *COM_D*). It can be understood as a penalty for non-fulfillment or partial compensation to the cheated party.

**Table 1:** The adapted game structure allowing commitment mechanisms.

Player A \ Player B	Com-coop (COM_C)	Cooperate (C)	Defect (D)	Com-defect (COM_D)	Com-only (COM_O)
Com-coop (COM_C)	R−e/2,R−e/2	*R*–*e*,*R*	–*e*,0	S+w−e,T−w	*R*–*e*,*R*
Cooperate (C)	*R*,*R*–*e*	*R*,*R*	*S*,*T*	*S*,*T*	*S*,*T*
Defect (D)	0,–*e*	*T*,*S*	*P*,*P*	*P*,*P*	*P*,*P*
Com-defect (COM_D)	T−w,S+w−e	*T*,*S*	*P*,*P*	*P*,*P*	*P*,*P*
Com-only (COM_O)	*R*,*R*–*e*	*T*,*S*	*P*,*P*	*P*,*P*	*P*,*P*

**Strategy Descriptions and Payoff Implications**

- ***C*** refers to the player choosing to cooperate without any additional conditions or mechanisms.
- ***D*** refers to the player acting purely in self-interest and does not cooperate.
- ***COM_C*** refers to the action of making a commitment first and then fulfilling it with cooperative behaviors. This strategy entails paying the commitment cost *e*. If both players use *COM_C*, they share this cost (each pays *e*/2), so both receive R−e/2. Against other strategies, a *COM_C* player often bears more of the commitment cost but still behaves cooperatively. For example, when a *COM_C* player encounters a pure defector *D*, the *COM_C* individual incurs only a small commitment cost and receives no additional payoff or punishment. Since *D* directly rejects cooperative engagement, no further rewards or penalties are distributed among the parties.
- ***COM_D*** makes a commitment but never fulfills it, which is more or less antisocial. When a *COM_C* player faces a *COM_C* player, the *COM_D* player may gain near-*T* but must subtract *w* for violating the commitment promise. Meanwhile, the *COM_C* player may partly recoup a portion *w*, even though they receive something close to the sucker’s payoff *S*.
- ***COM_O*** means one will never cooperate unless it involves a commitment. This strategy differs slightly from the others. While conventional prisoner’s dilemma assumes agents behave simultaneously, *COM_O* allows some implicit sequential decisions. Here, agents using *COM_O* may wait a short period to observe the other party’s action, then decide their action accordingly. Such a strategy enhances the model’s ability to capture sequential decision-making. We do not break it into two separate actions because observation itself does not yield any changes to the payoff.

Thus, the extended game structure allows us to describe and analyze interactions among agent. Agents could use the payoff structure to evaluate their expected payoff and decide which strategy to use. To sum up, it keeps the expressiveness while providing simple abstractions to the real-world phenomena in online social interaction, therefore setting up the foundations for our ABM.

#### Bounded rationality.

When people make decisions, their rationality is limited in many real-world situations, including deciding if to behave cooperatively in social interactions. As Young [19] pointed out, assuming an agent to have unlimited capability to obtain and process all information without making mistakes is super-rational rather than rational. To achieve the necessary fidelity of the model in describing social realities in social dilemmas, we took two measures to realize agents’ bounded rationality following the conventions [19]. The first is to limit the history that agents could use in decision-making, e.g., agents may only use the last five rounds of their own and their opponent’s game plays as the references in making decisions for the current round. The second is to let agents make random strategical decisions with a small percentage, e.g., 5%, to simulate the situations in which agents may make mistakes in choosing behaviors.

#### Propensity to cooperate.

The last theoretical lens used in our ABM is individual propensity to cooperate, which is indeed also a special case of bounded rationality. In a prisoner’s dilemma, a completely rational player’s strategy would be *defect*. But in the real world, “human beings can be anything but rational..,” as Lester Lave commented [41]. Some people always cooperate no matter what happens, while some always defect. In real-world settings, to cooperate or to defect,—is never a simple rational choice resulting from evaluating immediate gains [19].

We extracted this information from real-world experiments of prisoner’s dilemma. Jones [42] surveyed 36 studies of real-world experiments of prisoner’s dilemma from 1959 to 2003 and found that the median rate of cooperation is 39%, with a 19% minimum and an 80% maximum. Jones’ results suggest that people strongly prefer to *cooperate* or strongly prefer to *defect* would be less likely to lower than 20%. Recent studies in the lab and natural settings [43] also suggest similar results. So, when we model individual preferences, we would bring these insights into the model by allowing the initial distributions of individual strategies to fall into proper intervals. In our work, we break an individual’s payoff into two parts: the idiosyncratic payoff resulting from satisfying individual preferences and the interaction payoff received from interacting with another agent.

### Agents: How they make decisions and behaves?

With the above theoretical foundations, we could develop processes and rules to describe individual agent’s decision-making and behavior.

#### Formal specifications.

**Fig 1:**
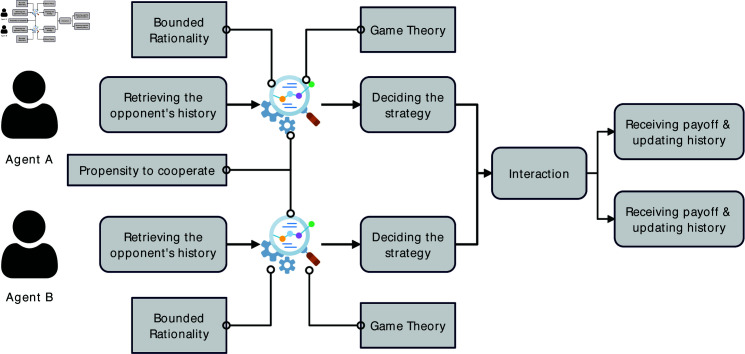
A simplified interaction scenario between two agents featuring their decision-making process.

Fig 1 describes a simplified interaction scenario between two agents. In this scenario, agents *A* and *B* are chosen to interact with each other.

In this process, agents first retrieve their opponents’ historical interaction information and use it as the basis for their decision-making. An agent’s decision-making is regulated by the decision framing [44] formed by the aforementioned theoretical foundations. Meanwhile, the **bounded rationality** limits agents’ capability in retrieving and processing information. Thus, only a part of the history of one’s opponent’s behaviors, rather than the entire history, could be used in the decision-making. Here, we allow an agent to only use the opponent’s latest *m* interactions as the references for the decision-making.

Now, we are going to incorporate different individual preferences. The total payoffs include idiosyncratic payoff resulting from one’s individual preference and interaction payoff ($P_{interaction}(s,\_s)$ in Eq 1 resulting from interacting with another agent under the game specified in Table 1. Doing so allows us to express the individual’s **propensity to cooperate** as a part of an agent’s utility. Supposing an agent *A* uses strategy *s*, the opponent uses *_s*, the agent’s payoff is:

$$U_{A}(s,\_s)=f_{A}(s)+P_{interaction}(s,\_s)$$ (1)

where *f*_*A*_(*s*) is a personalized function defined over the five strategies to represent the impact of an individual’s preference in cooperation on one’s utility.

When the agent *A* plays strategy *s*, agent *B*’s counter strategy *_s* could be one of the five strategies. Since the history (of size *m*) gives a possible distribution of *_s* over all the five strategies, let us use *k*_1_ to *k*_5_ to represent the frequency of a specific strategy in agent *B*’s history. An agent’s expected payoff of using strategy *s* can be written as Eq 2:

$$EP_{A}(s,\_s)=\frac{k_{1}}{m}\times U_{A}(s,COM\_C)+\frac{k_{2}}{m}\times U_{A}(s,C)+\frac{k_{3}}{m}\times U_{A}(s,D)\;+\frac{k_{4}}{m}\times U_{A}(s,COM\_D)+\frac{k_{5}}{m}\times U_{A}(s,COM\_O)$$ (2)

Given that we assume our agents are reasonably rational, they would choose the strategy *s’* that *maximizes* the expected payoff in the interaction with agent *B*. Then, the agent would behave accordingly to turn the decision into behavior. The agent *B*’s decision-making is identical, but with the agent *A*’s history as the reference. Obviously, their decisions are essentially the “*best-replies*” to each other [19]. However, in the real world, people often cannot calculate the expected payoffs precisely as we do above. They may only have some ambiguous guesses about the payoffs. Therefore, their decisions are often not deterministic. I.e., they may believe that it is possible that strategy X yields a better payoff than Y does, but they are not 100% sure about that. To describe the impact of ambiguity in decision-making, we follow the conventions and use the logistic learning rule [45] to specify the probability of choosing strategies. For a specific strategy *s*_*i*_ (1≤i≤5), the probability for the agent *A* to use it is Eq 3:

$$\frac{e^{EP_{A}(s_{i},\_s)}}{\sum_{i=1}^{5}e^{EP_{A}(s_{i},\_s)}}$$ (3)

Thus, our agent could accommodate the real-world uncertainty in decision-making. In this setting, if a strategy is likely to yield a higher payoff, it would be more likely but not definitely to be chosen as the strategy in the coming interaction. To turn the probabilities into a deterministic strategy in an interaction, the ABM utilizes the urn randomization technique [46] to select the strategy. Moreover, in some cases, agent *A* may not be that calculated, i.e., the bounded rationality may lead to mistakes in decision-making at a small but non-zero probability. We use θ to denote the probability of making mistakes.

### Collective dynamics

Developing cooperation might require multiple rounds of interactions. Therefore, our ABM model shall accommodate discrete-event feature for the purpose of analyzing long-term dynamics. During each round, two agents are randomly selected to interact. They follow the decision-making process described above to make decisions and interact. By continuing simulating interactions, the collective dynamics at the community level can automatically emerge and analyzed.

### Summary of the ABM model & its implementation

By now, we have built the main component of the model–an individual agent. Based on theoretical foundations, we defined how agents with diverse individual preferences make their decisions and behave in interactions in §4.2. Individual agents’ interactions form the collective dynamics. The next step is to implement the agent model and the process controlling the dynamic simulation process. The model is implemented with Python’s *Mesa* [47] framework for agent-based modeling. An agent is implemented as a class that encapsulates agents’ personal propensity to cooperate, decision-making, and behavioral history. Following the aspect-oriented programming logic, we use an independent process to deal with the concerns cross-cutting the entire simulation. The process controls and monitors the entire simulation process, including initializing the simulation, selecting participating agents in each period, tracking the periods, and monitoring and logging the entire simulation during the predefined number of periods (1,000 in our study, see Table 2. The source code of the ABM model implementation is publicly available at: https://figshare.com/articles/software/code/26095114.

**Table 2:** The list of parameters and their default values.

Parameter		Description	Default Value
Social Dilemma Payoffs	*T*	An agent’s payoff when playing *D* against *C*.	1.0
	*R*	An agent’s payoff when playing *C* against. *C*.	0.6
	*P*	An agent’s payoff when playing *D* against *D*.	0.2
	*S*	An agent’s payoff when playing *C* against *D*.	0.0
Commitment Setups	*e*	Cost of making a commitment.	0.1
	*w*	Penalty to defect after a commitment.	0.5
Individual Differences in Propensity to Cooperate		Strongly prefer to cooperate (20%).	[0.6, 0.9, -0.6, -0.9, 0]*
		Moderately prefer to cooperate (20%).	[0.2, 0.5, -0.2, -0.5, 0]
		Neutral (20%).	[0, 0, 0, 0, 0]
		Moderately prefer to defect (20%).	[-0.2, -0.5, 0.2, 0.5, 0]
		Strongly prefer to cooperate (20%).	[-0.6, -0.9, 0.6, 0.9, 0]
Bounded Rationality	*m*	The number of past interaction agents can observe.	5
	θ	Probability of making random decisions.	0.05
Simulation Settings	*N*	The population size of the agents in a simulation.	50
	*I*	The number of the interactions simulated in a single simulation run.	1,000

**Note:** *The idiosyncratic payoffs corresponding to strategies [*COM_C, C, D, COM_D, COM_O* ], similarly hereinafter.

## Simulation experiment design

The ABM described above allows us to explore several issues around individual preferences, commitment, and cooperation in the extended social dilemma through simulation experiments. In this section, we are going to introduce these experiments in detail. The experiment design is guided by the three main research questions. We thereby design two experiments to answer the $RQ_{1}$ and $RQ_{2}$ accordingly. To answer $RQ_{3}$, we conduct further analysis to experiment 1’s results.

### Experiment 1 for $RQ_{1}$

Since we want to check if individuals’ diverse preferences have any effects, we shall compare two conditions with and without considering individual preferences. Thus, simulations without individual preference could be considered as the “*control*” group, while simulations with individual preferences serve the “*treatment*” group. The only difference between the two groups is the presence of individual preference in cooperation. One of ABM’s methodological advantages is isolating other factors, so cross-comparisons between two groups would enable us to answer the $RQ_{1}$.

#### Experiment process.

**Fig 2:**
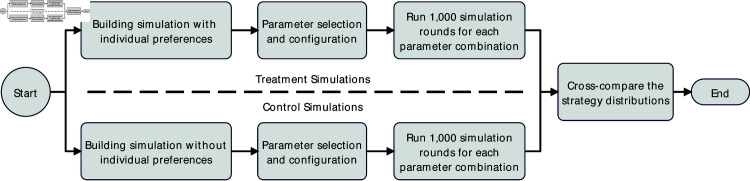
The overall process of the experiment 1.

Fig 2 describes the design of the experiment. We first build simulations for both conditions. They are basically the same, except that the treatment uses the payoff structure consisting of both idiosyncratic and interaction parts, while the control only has the interaction part. Then, we select simulation parameters based on prior literature and empirical evidence. Of course, in the treatment condition, we also need to determine the extra parameters related to individual preference in addition to parameter combinations in the control condition. After this step, we run 1,000 rounds of simulations for each parameter combination under two conditions. This study aims to measure changes in the proportions of the five strategies (*COM_C*, *C*, *D*, *COM_D*, and *COM_O*). While individual agents dynamically adjust their strategies during interactions based on the strategies they encounter within the population, our focus remains on the overall changes observed at the population level. Specifically, we measure the aggregated proportions of the five strategies across multiple simulation rounds, disregarding individual-level fluctuations. Once all simulations are concluded, we cross-compare these distributions to determine whether there are significant differences between the two conditions.

#### Experiment parameters.

In this experiment and the following two experiments, the parameters are determined following several simple heuristics. First, parameters should satisfy the quantitative relationships and other necessary conditions specified in related theories. For instance, a social dilemma requires *T* > *R* > *P* > *S*. Some specific relationships identify some critical boundary conditions. We reuse such boundary conditions to guide our experiments. Second, we leverage the rich empirical literature to set reasonable parameters. For example, the initial composition of agents could be determined by the evidence from many empirical prisoner’s dilemma experiments and their meta-analysis [42]. Third, setting the parameters should also follow common sense. For example, it might not be proper to allow magnitudinal differences between one’s idiosyncratic payoff and interaction payoff. Finally, to avoid excessive computations due to trivial numerical differences, we set the minimal difference in a quantitative relationship as 0.1.

Bearing the above in mind, we set the following default values to the parameters (Table 2). The first set of parameters is the payoffs in the classic social dilemma game (rows 1–4). We set parameters to satisfy the quantitative relationships among them. We let the maximal potential payoff (*T*) be “1”, and the minimal be “0.” Thus, all other payoff-related parameters could be proportions of “1.” The second set of parameters is the setup of the commitment (rows 5 & 6). Obviously, the cost of making a commitment should not exceed the payoff received from the cooperation. Since we have *R* = 0.6, *e* shall be less than 0.6, thus in the range 0.1 to 0.5. Given the voluntary nature of most collaborative activities that involve commitment mechanisms, the penalty *w* for failing to honor the commitment should not be excessively high. So, we assume it is in the same range as the cost. In the default setting, we set *e* and *w* as 0.1 and 0.5, respectively. In experiment 2, we manipulate them to examine the impact of different setups of commitment. The third class of parameters is related to individuals’ idiosyncratic payoffs resulting from their own propensity to cooperate (rows 7–10). First, according to Jones’ and other empirical experiments of prisoner’s dilemma, those who stick to cooperate or defect are usually about 20% each in the whole population. So we let those who strongly prefer to cooperate (or defect) account for 20% of all agents. The remaining 60% are also equally divided to simplify the computation and randomization (moderately prefer to cooperate, neutral, and moderately prefer to cooperate). Then, to avoid potential violations of the von Neumann–Morgenstern utility axioms, the maximal idiosyncratic payoff shall be less than the potential maximal payoff from interacting with others. So, we let the payoff for “strongly prefer...” and “moderately prefer...” be 0.9 <1.0 = *T* and 0.5 <0.6 = *R*, respectively. The averse options’ payoffs are the additive inverse, e.g., –0.9 for *COM_D* for an agent who strongly prefers to cooperate. Meanwhile, the difference to the next choice is 0.3. The last set deals with one’s bounded rationality (rows 11 & 12). We let one could only use the last 5 rounds of the opponent’s strategies in making decisions (*m* = 5) and making random decisions (mistakes) at the probability of 0.05 (θ). We set the size of the agents’ population as 50. Besides, please note that these numerical values of parameters in Table 2 are also applied to the following simulation experiments. The default values would remain as they are unless explicitly specified.

#### Simulation initialization.

The simulation is initialized with 50 agents. In the *Control* condition, agents are set to play with uniformly-random strategies at the beginning because there is no history available as the reference for them to practice the decision-making specified in §4.2. Since there are five strategies, randomly selecting strategies would result in a state that every strategy starts with 20%. Once the history is established, agents make decisions using the process described in §4.2. The initial states are different in the *Treatment* condition. We let agents use an initial strategy to match their propensity to cooperate most, i.e., the strategy yields the highest idiosyncratic payoff. As shown in Table 2, agents who strongly and moderately prefer to cooperate or defect choose *C* and *COM_D*, respectively. For agents neutral to cooperate, their initial strategy is uniform sampled from all strategy. According to the population setups in Table 2, about 44% (40% + 20% × $\frac{1}{5}$) agents’ initial strategy is *C*. Similarly, 44% agents’ initial strategy is *COM_D*. The proportions of agents starting with strategies *COM_C*, *D* or *COM_O* are all about 4%. By default, the initial states in the *control* and *treatment* groups differ due to the manipulations introduced in the *treatment* condition. However, this difference does not pose a threat to the validity of the conclusions, as it is an inherent aspect of the experimental design. To address this, we modify the control group to ensure the initial strategy distribution identical to that of the treatment group, excluding considering idiosyncratic payoffs.

### Experiment 2 for $RQ_{2}$

Prior literature shows that commitment is an effective mechanism to carry social dilemmas to cooperation under certain setups of the commitment [11, 30]. These setups are often specified in the quantitative relationships between the cost of commitment (*e*) and the penalty (*w*) to those who commit but not deliver. One such theoretical condition is given in Han *et al*. [11], where they claim that when $e<\frac{2R}{5}$, and $w>max\{\frac{T-R-S}{2}\;-\;\frac{3e}{4},\frac{T-R-2S}{3}\;+\;\frac{5e}{6}\}$, the cooperation is more likely to be achieved. Since our second research question is to examine the effects of different setups of commitment when introducing individuals’ propensity to cooperate, the second experiment would use such theoretical results to guide our experiment.

**Fig 3:**
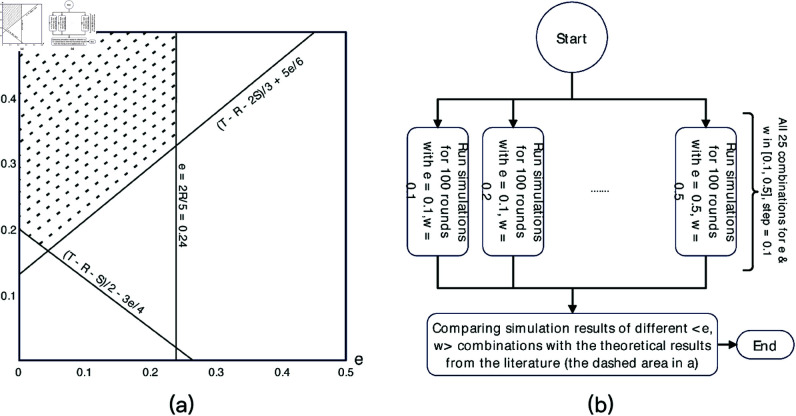
The plots of the theoretical results from Han *et al*. (2012) and the overall process of the experiment 2.

With the default parameter values, the condition in Han *et al*. is *e* < 0.24, and w>max{0.2 − $\frac{3e}{4},0.13\;+\;\frac{5e}{6}\}$. If plotting them in a coordinate, the condition defines an area marked with dashes in Fig 3a. This area is mostly in around the upper left corner of Fig 3a, where the cost of commitment is small but the penalty is large. According to Han *et al*., [11], when <*e*,*w* >  falls into this area, it would be more probable for a large proportion of individual agents to use strategies leaning toward cooperate (*C* and *COM_C*). If such a condition still holds when considering diverse individual propensity to cooperate, we could use it in the design of commitment mechanisms. Experiment 2 is designed to verify this.

Fig 3b describes the process of the second experiment. In experiment 2, we do not need the control and treatment conditions. Instead, we let the parameters *e* and *w* vary in the range [0, 0.5] with a minimal step size of 0.05 (see §5.1.2), resulting in a total of 121 combinations of <*e*,*w* > , ranging from <0.0,0.0> to <0.5,0.5>. We then run 1000 rounds of simulations with all these 121 combinations. For each combination, we compute the average proportion of agents using strategies leaning to cooperation when their behaviors become stable. By comparing such proportions under the 121 combinations, we can check if the theoretical results still hold when considering individual preferences.

### Further analysis for $RQ_{3}$

We are going to provide answers to $RQ_{3}$ by focusing on the process of the dynamics of cooperation in social dilemmas interactions. It would not need to run any new simulations. Instead, it performs further quantitative analysis towards the simulations obtained in answering the $RQ_{1}$. This analysis focuses on the trajectories of the simulation results. Since we have five strategies, there are two possible cases after 1,000 periods in a simulation.

**Case 1** : The proportions of individuals using the five strategies become stable. Most people stick to a specific strategy and seldom change their strategies.

**Case 2** : The proportions of individuals using the five strategies still keep changing. Most people switch strategies in their interactions.

**Fig 4:**
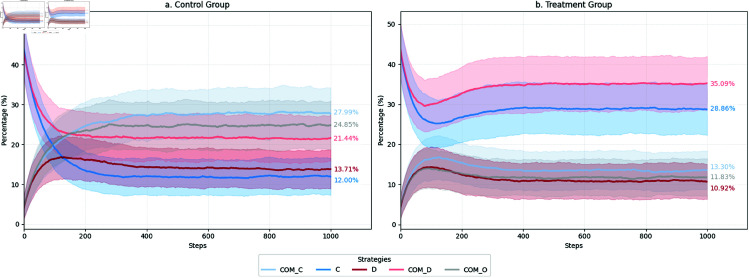
The dynamics of strategies under two experiment conditions.

Theoretically, the first case means simulations enter some absorb states. In fact, our simulations do exhibit such a characteristic (see Fig 4). Simulations in both *Control* and *Treatment* conditions become stable in the later phases of the 200 simulated periods. Therefore, we would only need to deal with the **Case 1** and ignore the **Case 2** in our analysis. For this type of trajectory, we could analyze the efficiency and stability by defining a critical period *t*_*c*_. For any period before it, the variances of a strategy’s proportions are larger than the specific value *c*, and for any period after it, the variances of the strategy’s proportions are smaller than the specific value *c*. Supposing we let c=2%, we could say before a specific $t_{2\%}$, the process of strategy *s* is not quite stable at the criterion of 2% variances but becomes very stable after it. Since we have five strategies, once all strategies’ dynamic trajectories arrive *t*_*c*_, the whole simulation becomes stable. In the analysis, we calculate the *t*_*c*_ for both conditions and compare them.

### Sensitivity analysis

For each experiment, the corresponding sensitivity analysis is performed. We perform two classes of sensitivity analysis. The sensitivity analysis focuses on the four parameters in the payoff structure (see Table 2). In general, the game requires a quantitative relationship that *T* > *R* > *P* > *S*, which allows a wide range of values for these parameters. In the sensitivity analysis, we manipulate their values while keeping their relative quantitative relationships to examine if variances in the basic game influence our results. The second class of sensitivity analysis deals with different settings of individual preferences in cooperation. In our experiment, we have a 5-tier arrangement of individual preferences, i.e., *strongly prefer to cooperate*, *prefer to cooperate*, *neutral*, *prefer to defect*, and *strongly prefer to defect*. Each accounts for 20% of the whole population. We have shown that the prior empirical evidence supporting the manipulation of assigning 20% individuals into *strongly prefer to cooperate* and *strongly prefer to defect*, respectively. However, the 60% individuals in the middle may not be evenly distributed. In the sensitivity analysis, we test three other different arrangements: (1) all 60% are neutral; (2) 40% *prefer to cooperate*, 20% *prefer to defect*, and no neutral; and (3) 20% *prefer to cooperate*, 40% *prefer to defect*, and no neutral. Note that the sensitivity analyses are supplementary to the main experiments. We put it in Appendix rather than including its details in the next section unless they form some severe challenge to the findings.

## Results & findings

This section reports on the results of our study. Note that all simulations have been running multiple rounds in our experiments, so the results are aggregated averages of all simulation rounds rather than a single run of each simulation. Repeating simulation multiple times enables us to get converged results rather than random errors resulting from a single simulation run [36].

### Experiment 1 results & findings

In experiment 1, we attempt to compare the dynamics of cooperation under two conditions: with and without considering individual preferences in cooperation. Fig 4 describes the aggregated dynamics of agents’ behavior distributions over time. In each plot, the *x*-axis represents the periods over time; the *y*-axis represents the percentage of agents using a specific strategy.

First, let us have a look at what happens under the *Control* condition (Fig 4a). In this condition, the system initializes at the even distribution of the five strategies. Since agents have no preferences and no history for them to make calculated decisions, they play randomly at the very beginning (see §5.3.7 for details of simulation initialization). Later, we can see some strategies gain popularity among agents while others lose. Eventually, each strategy’s share becomes stable after hundreds of interactions. *COM_C* becomes the strategy adopted by most individuals in the long run. Roughly 28% of agents would use it. *COM_O* is the second most popular one, which is used by 25% of agents. The third popular strategy is *COM_D* (22%). The fourth is *D*, and the fifth is *C*. They account for about 14% and 12% of agents, respectively.

Fig 4b shows the dynamics under the *Treatment* condition. Note that the initial phase is different since people tend to play their favorite strategies at the beginning when considering their individual preferences (see §5.3.7 for details of simulation initialization). The long-term strategy choices are different. *COM_D* is the most popular condition, which is played by about 35% of agents. *C* holds the second place with about 29% of agents using it. The other three strategies are adopted by a similar amount of agents, which are 14% for *COM_C*, 12% for *COM_O*, and 11% for *D*.

Obviously, considering diverse individual preferences does have some effects. Regardless of the differences in the early simulation initialization phase, there are several critical differences in the long-term dynamics. Table 3 summarizes these differences. They indicate that the commitment mechanism’s effects in promoting and maintaining cooperation are undermined. The most uncooperative and antisocial strategy (*COM_D*: making commitments but never fulfilling them) becomes the top choice. While about the same amount of people choose to be cooperative by playing *COM_C* and *C*, much more people lean to be uncooperative unconditionally (*COM_D* + *D*: 46% vs. 36%). The conditional cooperative individuals (using *COM_O*) are out of the game (25% vs. 12%). However, commitment still has some positive effects by preventing those who prefer to cooperate from adopting uncooperative strategies. Note that the number of agents who use *C* is much higher under the treatment condition. It helps maintain the bottom-line cooperation in a community.

**Table 3:** The proportions of each strategies under two experiment conditions.

	*Control*	*Treatment*
Popularity	COM_C>COM_O>	COM_D>C>
	COM_D>D>C	COM_C>COM_O>D
*COM_C*	≈ 28%	≈ 14%
*COM_C* + *C*	≈ 40%	≈ 43%
(Lean to cooperate)		
*COM_D*	≈ 22%	≈ 35%
*COM_D* + *D*	≈ 36%	≈ 46%
(Lean to defect)		

Based on the above results, we can answer the $RQ_{1}$ as follows:

*The commitment’s positive effects on promoting cooperation are undermined but still exist. The amount of individuals who take cooperative strategies remains at a similar level (43% vs. 40%), but more individuals may choose the antisocial strategy and lean to defect unconditionally (36% vs. 22%). In general, commitment’s effects are exhibited in the form of preventing people who prefer to cooperate from taking uncooperative strategies.*

### Experiment 2 results & findings

**Fig 5:**
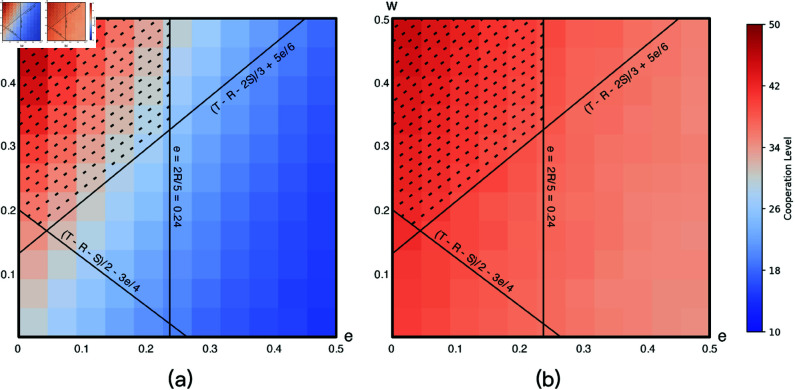
The average proportions of individual agents using strategies leaning to cooperation under different setups of commitment.

With Fig 3a as the background, we use a heatmap (Fig 5) to visualize the average proportions of individual agents using strategies *C* or *COM_C* under the 121 combinations of <*e*,*w* > . The heatmap is divided into two panels, representing the control group and the treatment group, to illustrate the differences in cooperation levels when individual preferences are excluded or incorporated.

In the control group (Fig 5a, where individual preferences are not considered, cooperation levels primarily relies on smaller commitment costs and higher penalties (upper-left region). Conversely, in settings with high commitment cost *e* and low penalties *w*, cooperation levels drop significantly. These results align closely with prior literature such as Han *et al*. [11], indicating that cooperation levels in homogeneous populations are sensitive to the parameter settings of the commitment mechanism.

In the treatment group, the results are straightforward. The top left corner (*e* = 0 and *w* = 0.5), which also indicates the combination of smallest cost of commitment and highest punishment, records highest proportion of agents using *C* or *COM_C* (45%). The proportions gradually decrease in both directions until the bottom right corner, where 34% of agents use *C* or *COM_C*. From Fig 5b, it is easy to conclude that the effects of different setups of commitment are in a similar direction with control group. However, recall that the simulations initialize from a situation where about 44% of agents prefer to cooperate when considering individuals’ diverse preferences. Even the best case with the commitment <0.1,0.5> has a 1% increase (44% vs. 45%) in the proportion of agents who play cooperative strategies. Notably, the introduction of individual preferences leads to an overall increase in cooperation levels across various configurations of the commitment mechanism. Specifically, even under conditions of higher commitment costs (*e* > 0.2) and lower penalties (*w* < 0.3), cooperation levels remain relatively high, with a minimum of 34% observed. In this sense, the effect of the commitment mechanism is **not guaranteeing** a majority of members to be cooperative, but **preventing** members from changing to uncooperative ones, which is also consistent with $RQ_{1}$’s findings. This indicates that the heterogeneity among individuals enhances the collective resilience of cooperation, allowing the population to maintain cooperative behavior even under more relaxed enforcement mechanisms.

Therefore, we can answer the $RQ_{2}$ as follows:

*Different setups of the commitment mechanisms and individual preferences play complementary roles in shaping cooperation levels. This indicates that the primary effect of the commitment mechanism is not to guarantee a majority of agents adopting cooperative strategies, but rather to prevent cooperative agents from turning uncooperative. Furthermore, the heterogeneity among individuals strengthens the collective resilience of cooperation, reducing reliance on strict external enforcement and allowing for stable cooperation under more relaxed conditions.*

### Results & findings of the analysis for $RQ_{3}$

In this analysis, we would like to study the efficiency and stability of the commitment’s impacts on cooperation with individual preferences. As the design of the analysis indicates, we could calculate an important period *t*_*c*_ to characterize the efficiency and stability of each strategy. We set c=5%. Since we have a population of 50 agents, c=5% means no more than two individuals switch strategies at any time. Table 4 summarizes the *t*_*c*_s of each strategy under both *Control* and *Treatment* conditions. It seems there are not many differences between both conditions. The *Treatment* condition reaches stable states slightly faster than the *Control* condition (529 vs. 625). However, it is still a substantial amount of time. Given that we have 50 agents and each interaction requires two agents, 529 periods of interaction mean that each agent has already participated in more than 21 interactions on average. In the real world, 21 interactions in a community may take months, while many participants’ entire careers in a community are often several months [48]. Hence, future research may need to explore possible interventions that could potentially shorten this process.

**Table 4:** The *t*_*c*_s of each strategies under both *Control* and *Treatment* conditions.

Strategy	*Control*	*Treatment*
*COM_C*	523	461
*C*	360	397
*D*	625	529
*COM_D*	196	335
*COM_O*	287	392

Therefore, we can answer the $RQ_{3}$ as follows:

*The Treatment condition reaches stable states slightly faster than the Control condition, yet still takes a substantial amount of time. On average, an agent may need to participate in dozens of interactions before the whole system’s dynamics become stable.*

### Summary

By now, we have provided answers to all three research questions. The corresponding sensitivity analyses are also performed (see Appendix). We do not identify any significant challenge to the main results and findings. To keep the paper concise, we decide not to include detailed results of sensitivity analyses. The next section will discuss the findings, their theoretical, methodological, and practical implications, and the limitations of the study.

## Discussion

### Discussion of findings

Our extensive experiments yield rich findings, which could turn into insights about cooperation in extended social dilemmas. First of all, experiment 1’s results suggest that the commitment itself, though insufficient to make the whole population cooperative, does help maintain bottom-line cooperation in the community consisting of members of diverse preferences. Its positive effect lies in preventing cooperative people from taking uncooperative behaviors. Second, experiment 2’s results show that proper setups of commitment mechanisms are still useful in maintaining cooperation. In real-world scenarios, such as collaborative or exploratory communities, organizations may consider strategies to reduce the cost of commitment while increasing penalties for noncompliance to enhance the effectiveness of commitment mechanisms. Then, the $RQ_{3}$’s results show that it is difficult for a community to reach stable states no matter whether their members have diverse preferences, raising the necessity of designing proper interventions to shorten such a process. Moreover, from an individual perspective, members may need more time to stabilize their own strategy, spending extra efforts in “*trial and error*.” Lastly, it is surprising to find that antisocial players who take the strategy *COM_D* significantly increase in the population (22% vs. 35%). Such an increase may result from two reasons. First, heterogeneous preferences encourage *COM_C* players to switch to *C* players and lead to more initial *COM_D* players. Therefore, the antisocial players are less likely to meet *COM_C* players, in which they could be punished. So, playing their favorite strategy *COM_D* becomes much safer. They have no incentive to change their favorite strategy. Second, idiosyncratic payoffs create some basin of attraction around *COM_D*, which makes it hard for some agents moderately prefer defect to escape once they accidentally enter it. Besides, switching strategies (see Eq 3) are not deterministic but probabilistic, prohibiting an agent from escaping from that basin of attraction.

To sum up, by introducing diverse individual preferences, our ABM could help derive deeper and more realistic understandings and insights towards cooperation dynamics in addressing social dilemmas. Thus, our study has multiple implications for theory development and potential practical applications in future scenarios.

### Theoretical implications

As an instance of emerging social collaboration analytics [49], our work’s theoretical implications are tri-fold. First, it extends the theoretical knowledge of the commitment mechanism’s role in facilitating cooperation. Researchers such as Han *et al*. [11, 30] have built solid theories of commitment’s role in the emergence and development of cooperation. However, neglecting individual preferences in cooperation restricts their theories’ capability to describe and explain real-world phenomenon and prescribe solutions for real-world problems. Our work fills such a theoretical gap. Moreover, our results and findings confirm that introducing diverse individual preferences is of high necessity to enhance our understanding of the intricate interrelations between commitment and cooperation in social dilemmas. The above discussion of findings makes our work’s theoretical value evident. Second, our work also features the innovative use of extant theories and empirical evidence to inform the design of ABM (See Fig 1). Doing so enables us to take advantage of multiple theories’ in modeling agents’ complex decision-making, as well as achieving high fidelity in describing realities. Also, the architecture of the ABM design leaves space for integrating other theories and empirical evidence in the future. Third, our work also opens rich future opportunities. For example, we have not yet considered the social network formed by individuals, which could be an immediate enhancement to our work. Besides, the details of strategy switching have not been studied, though we have the data ready. Meanwhile, it is also important to validate our theoretical findings in empirical settings either by analyzing the digital traces of real-world scenarios or conducting controlled lab experiments with human subjects. These future studies could build a holistic and realistic knowledge body of commitment and cooperation in various social dilemmas scenarios.

### Practical implications

By introducing diverse individual preferences into the interrelations between commitment and cooperation, our work has immediate implications to practitioners of social dilemmas, particularly the decision-makers.

First, our results emphasize the importance of considering individual preferences in the development of cooperation, thus informing decision-makers to properly evaluate their commitment mechanisms’ effectiveness by considering members’ heterogeneous preferences. The recent flourish of automated preference inference techniques, e.g., Houlsby *et al*. [50], significantly reduces the cost of performing such evaluations. Decision-makers could reuse our ABM model and combine it with the individual preferences inferred from members’ digital traces to run mechanism evaluations before launching in their communities.

Second, our results demonstrate the importance of articulating the setups of commitment mechanisms. Practitioners may navigate and explore the design space of the commitment mechanism following two directions, i.e., reducing the cost of commitment and increasing the penalty to those who make commitments but never fulfill them. The former may be realized by automated tools matching tasks with particapants’ information and capability so that they could spend less time and effort determining if they should commit. The latter could be realized through broadcasting defectors’ misconducts to a wide range of members in the community since that could drastically lead to significant reputation loss (as a form of penalty) [51, 52].

Third, as Clark argued, commitments are essential to all true joint activities while many individuals might commit themselves privately to doing something [53]. These individuals could be viewed as de facto cooperators from behavioral level. However, the existence of members who prefer *COM_O* means that private commitment might contribute to others’ uncooperative behaviors which are fairly stable in the long run (see Fig 4). Such uncooperative behaviors may further motivate those who commit privately to switch to less cooperative behaviors, which might lead to unfavorable output. Therefore, it might be necessary to make private commitment explicit in a low-cost way. Meanwhile, it should also avoid embarrassing people prefer to make commitment privately. In the example in the introduction, we can notice that a commitment in modern social cooperation often requires an individual to make explicit verbal statement in public. The future design may explore mechanisms and designs allowing certain form of indirect, less explicit public self-commitment by leveraging some nonverbal signals.

### Supporting decision-making in social collaboration

**Fig 6:**
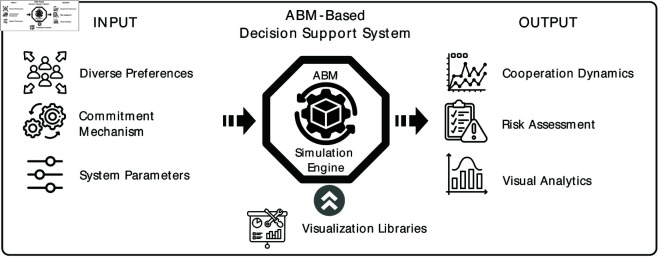
The overview of decision support system.

The agent-based model described in this paper forms the foundation of a fully-functional decision support system which could help decision-makers. Fig 6 shows the simulation engine of decision support system integrated the ABM model. The agent-based model can be initialized by the practical preference distribution, certain commitment mechanism and system parameters of specific social dilemma scenarios. Leveraging model simulation and visualization, the system could offer comprehensive insights to decision-makers for facilitating cooperation.

First, decision-makers can make informed decisions by analyzing the outcomes that reveal whether the cooperation in a community could reach stable states at the population level in the long run. For example, In scenarios where the simulation results become unstable, these outcomes may be caused by external shocks to the community, failure of commitment or other control mechanisms, and the growth of antisocial behaviors [6]. Monitoring such dynamics supports community maintainers in identifying potential risks and uncertainties, enabling them to decide whether to implement intervention or corrective action. Even in a stable but not ideal state, e.g., only a low percentage of cooperative behavior, the maintainer can optimize community management measures in case some antisocial behaviors trigger *tragedy of commons*.

Second, the system based on our model offers decision-makers a powerful tool to simulate and evaluate the commitment mechanism in different decision scenarios. These simulation scenarios can be created by integrating well-designed commitment mechanisms, modifying the governing rules or model parameters around real-world settings, or introducing external intervention. With extensive simulations, decision-makers enable to observe how these changes affect the system. Thus, they can effectively evaluate and optimize the commitment mechanisms to promote cooperation in their communities. Besides, decision-makers can conduct detailed risk assessments to identify potential abnormal changes and their impact on the community.

Third, the system could easily accommodate visualization interfaces to provide customized visual analytics for decision-making [54, 55]. The agent-based model is implemented in Python, making it easily coordinate with various Python visual analytics libraries, e.g., Plotly. Therefore, it could offers rich visualization interfaces to allow users to explore the dynamic process of community cooperation evolution over time. Leveraging the model-integrated visualization tools and techniques, such as time-series plots, phase diagrams, or Mesa [47], decision-makers can identify key agents and interactions within the system and trace the evolution of cooperative behavior in online social networks, enabling them to get deep insights before taking actions in an interactive fashion [56].

### Limitations

Our work has several limitations that shall be acknowledged. The key limitation is inherited from the methodological nature of the agent-based modeling. No matter how complex, a computational model is merely a simplified abstract of the real world. A human being is much more complicated than an agent who behaves according to predefined rules. The theories and empirical evidence integrated into our model increase our confidence in the results and findings but never guarantee 100% correctness. For example, the numerical values to parameters are likely to not fully capture the reality. However, such a fallible nature ensures our approach to be scientific [36], and gives us spaces for future refinement of our work. Another limitation is that our model is not directly tied to any specific real-world scenario. Instead, it builds upon and extends the theoretical framework proposed in Han’s studies [30] to investigate the dynamics of commitment mechanisms and individual preferences. We argue that the incremental way of investigating the important ones first makes our work more feasible and avoids losing in the complicated joint effects of many factors. Lastly, every decision in designing and implementing an ABM is a compromise. While we are confident in achieving good balance, our own bounded rationality may lead us to make suboptimal decisions. In addition, although using extended two-person prisoner’s provides significant convenience to describe the interaction among individuals, it loses certain advantages of modeling the whole system with a *n*-person public goods game. Such advantages include direct reasoning on collective actions and outcomes. Thus, relying on a single model may limit the study’s capability to abstract, which should be fixed in future research.

## Conclusion

This article reports on our agent-based modeling and simulation efforts for investigating the complicated interrelations between commitment and cooperation with special consideration of diverse individual propensity to cooperate. We combine multiple theoretical insights and empirical evidence to design the ABM and run extensive simulation experiments with it. Our results reveal that: when considering individual preferences, (1) commitment’s positive effects on promoting cooperation are undermined but still exist and exhibit in the form of preventing people of goodwill from taking uncooperative strategies; (2) different setups of commitment mechanisms still matter, and smaller cost of making commitments and larger penalty for failing to fulfill commitments are desirable; (3) it takes a substantial time for a community to achieve stable states, thus some interventions shorten the process would be helpful. Our results and findings have direct implications for research and practices aiming at improving cooperation in social dilemmas. We also discuss our model’s fundamental capability in supporting decision-making. For future work, we plan to continue enhancing the ABM model by trying to incorporate the *n*-person public goods game. This would allow the researchers to choose which base model to use according to their research settings and phenomena of interest. We will also build and evaluate a decision-making tool around the models.

## Appendix

### Results of sensitivity analysis

As we mentioned before, two series of sensitivity analyses are conducted. In this section, we summarize the results of these sensitivity analyses and report them accordingly.

#### Sensitivity analysis with different payoff values.

**Fig 7:**
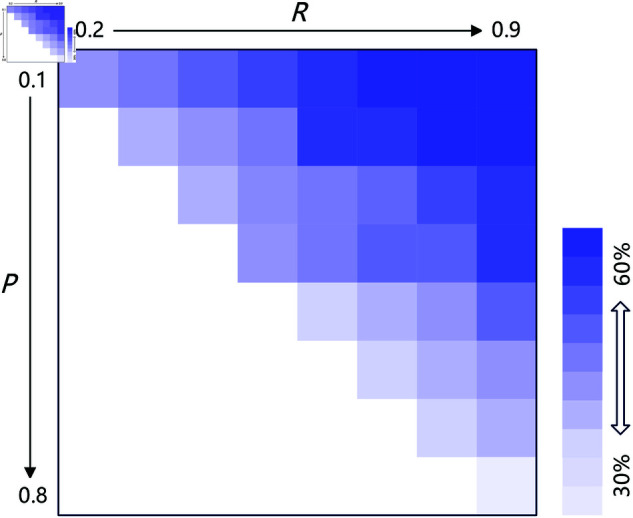
The proportion of individual agents lean to cooperate under different combinations of *R* and *P.*

Our game model requires a quantitative relationship that *T* > *R* > *P* > *S*; the first sensitivity analysis deals with the different payoff values. In this analysis, we keep *T* = 1 and *S* = 0 intact, and let *R* and *P* vary in [0.1, 0.9]. Fig 7 plots the results of the sensitivity analysis. In Fig 7, each cell represents a combination of*R* and *P*, and the grayscale in it represents the percentage of agents who would keep using lean to cooperate (using strategies *COM_C* and *C*) in the long run. In general, we can have two conclusions. First, no matter how the payoff parameter varies, there is still a substantial proportion of agents, at least over 40%, who keep using strategies lean to defect. Second, there are always more agents, at least over 10%, than those who strongly prefer to cooperate and keep using strategies lean to cooperate. Neither conclusion has material differences from the results in Results & findings.

#### Sensitivity analysis with different individual preference setups.

The second sensitivity analysis deals with different individual preference setups. In Simulation experiment design, we choose to use a five-tier, evenly distributed individual preferences system. Here, we manipulate the 60% agents’ preferences in the middle. As we mentioned above, there are three arrangements: (1) all 60% are neutral; (2) 40% *prefer to cooperate*, 20% *prefer to defect*, and no neutral; and (3) 20% *prefer to cooperate*, 40% *prefer to defect*, and no neutral. Table 5 summarizes the results as follows, where the original arrangement is used as the benchmark (arrangement 0). From Table 5, we can find that: (1) different arrangements of individual preferences lead to some variances in the distributions of agents’ choice of long-term strategies; however, (2) they are not powerful enough to eliminate the use specific strategies, no matter whether such strategies lean to cooperate or defect. Therefore, the results in Results & findings are still valid in principle.

**Table 5:** The proportions of each strategies under different arrangements of individual preferences.

	*A. 1*	*A. 2*	*A. 3*	*A. 0*
*COM_C*	≈ 28%	≈ 11%	≈ 30%	≈ 13%
Lean to cooperate	≈ 32%	≈ 51%	≈ 37%	≈ 42%
*COM_D*	≈ 22%	≈ 14%	≈ 41%	≈ 35%
Lean to defect	≈ 56%	≈ 38%	≈ 50%	≈ 46%
